# Effects of acids pre-treatment on the microbial fermentation process for bioethanol production from microalgae

**DOI:** 10.1186/s13068-019-1533-5

**Published:** 2019-07-31

**Authors:** Chai Kee Phwan, Kit Wayne Chew, Abdi Hanra Sebayang, Hwai Chyuan Ong, Tau Chuan Ling, Marlinda Abdul Malek, Yeek-Chia Ho, Pau Loke Show

**Affiliations:** 1 Institute of Biological Sciences, Faculty of Science, University of Malaya, 50603 Kuala Lumpur, Malaysia; 2 Department of Chemical and Environmental Engineering, Faculty of Science and Engineering, University of Nottingham Malaysia, Jalan Broga, 43500 Semenyih, Selangor Darul Ehsan Malaysia; 3 Department of Mechanical Engineering, Faculty of Engineering, University of Malaya, 50603 Kuala Lumpur, Malaysia; 4 Institute of Sustainable Energy (ISE), Universiti Tenaga Nasional, 43000 Kajang, Selangor Malaysia; 5 Civil and Environmental Engineering Department, Universiti Teknologi PETRONAS, 32610 Seri Iskandar, Perak Darul Ridzuan Malaysia; 6 Centre for Urban Resource Sustainability, Institute of Self-Sustainable Building, Universiti Teknologi PETRONAS, Seri Iskandar, Perak Darul Ridzuan Malaysia

**Keywords:** Bioethanol, Fermentation, Microalgae, Pre-treatment, Reducing sugar concentration

## Abstract

**Background:**

Microalgae are one of the promising feedstock that consists of high carbohydrate content which can be converted into bioethanol. Pre-treatment is one of the critical steps required to release fermentable sugars to be used in the microbial fermentation process. In this study, the reducing sugar concentration of *Chlorella* species was investigated by pre-treating the biomass with dilute sulfuric acid and acetic acid at different concentrations 1%, 3%, 5%, 7%, and 9% (v/v).

**Results:**

3,5-Dinitrosalicylic acid (DNS) method, FTIR, and GC-FID were employed to evaluate the reducing sugar concentration, functional groups of alcohol bonds and concentration of bioethanol, respectively. The two-way ANOVA results (*p* < 0.05) indicated that there was a significant difference in the concentration and type of acids towards bioethanol production. The highest bioethanol yield obtained was 0.28 g ethanol/g microalgae which was found in microalgae sample pre-treated with 5% (v/v) sulfuric acid while 0.23 g ethanol/g microalgal biomass was presented in microalgae sample pre-treated with 5% (v/v) acetic acid.

**Conclusion:**

The application of acid pre-treatment on microalgae for bioethanol production will contribute to higher effectiveness and lower energy consumption compared to other pre-treatment methods. The findings from this study are essential for the commercial production of bioethanol from microalgae.

## Introduction

Rapid growth of the world population and improved developments over the past decade have increased the demand for energy which is mainly derived from fossil fuels. This has led to severe environmental impacts such as the release of greenhouse gases (GHG) emission to the atmosphere, which is one of the major contributors to global warming and ocean acidification [1]. To overcome these issues, an alternative source of renewable energy is required to replace the depleting fossil fuels. Bioethanol is one of the potential alternative biofuels that can reduce the dependence on fossil fuels in the near future. It can be classified into first-generation bioethanol derived from agricultural crops; second-generation bioethanol that are mainly produced from lignocellulosic materials and third-generation bioethanol which are derived from microalgae [2, 3]. Bioethanol production from microalgae has many advantages over the first and second-generation biofuels due to its fast growth rate, ability to grow on wastelands for cultivation and does not pose food security issues [4, 5]. Moreover, microalgae cells lack lignin content which makes them disrupt easily compared to lignocellulosic materials, in addition to the cheaper operation cost than second-generation biofuels.

The growing rate of *Chlorella* biomass is relatively fast with an extrapolated productivity of 25 g/m^2^ per day and the composition of this species is about 40–70% of carbohydrates, 10–20% of protein and the residual low-molecular-weight compounds, which are fatty acids, free amino acids and amines [6]. Microalgae-based carbohydrates consist of mainly starch and cellulose with the absence of lignin, this makes it easier to break them down into monosaccharides and convert them into bioethanol. Microalgae biomass is a suitable biomass feedstock to produce large quantities of bioethanol due to its high content of carbohydrate [7, 8]. Furthermore, Zhou et al. [6] reported that the *Chlorella* sp. TIB-A01 species was capable of obtaining a sugar concentration of 12 wt% (v/v) and producing an ethanol yield of 0.47 g/g of sugars. Thus, *Chlorella* microalga was selected because of its capability with high proton efficiency to synthesise and convert a large quantity of carbohydrates into bioethanol production [9].

Pre-treatment is one of the crucial processes to breakdown the cell walls of the microalgae biomass to release fermentable sugars before converting them into bioethanol in the fermentation process. An efficient pre-treatment process takes into consideration the production cost, energy efficiency, ease of application, degradation capacity of fermentable sugars as well as the feasibility for commercialization [10, 11]. Diluted acid is considered as one of the inexpensive pre-treatment method that is widely applied. For example, pre-treatment method using dilute sulfuric acid has been reported to obtain a higher hydrolysis yield compared with hydrochloric, phosphoric, and nitric acid [11]. It has been known that starch is easily hydrolyzed with diluted acid in microalgae biomass. During acid pre-treatment, the acid concentration is one of the significant factors that can affect the release of the fermentable sugars and conversion yield [10]. Several studies of sulfuric acid pre-treatment have been shown to be an effective pre-treatment method for different species of microalgae biomass such as *Scenedesmus abundans, Dunaliella* sp., and mixed microalgae cultures. [12, 13]. Sulfuric acid is one of the most commonly used options because the anion (SO_4_^2−^) released during the pre-treatment process acts as the nutrient for fermenting yeast [14]. However, the use of this strong acid can result in corrosion of equipment and compromise on the final product quality, where these drawbacks could be avoided with the use of weaker acids.

This work focuses on the effects of different types of acid with varying concentrations on the release of fermentable sugars to be further converted into bioethanol through fermentation process. The bioethanol product quality was evaluated based on the reducing sugar concentration, functional groups of alcohol bonds and concentration of bioethanol. This study contributes to the development of a cost-effective, energy saving and commercial outlook for the efficient bioethanol production from microalgae in the future.

## Materials and methods

### Materials and substrates preparation

The substrate used in this study was microalgae *Chlorella* powder obtained from Korea. 10 g of substrate was weighed and poured into SIMAX-200 ml glass reagent bottles with cap. Enzyme α-amylase from *Bacillus licheniformis* Type XII-A with an enzymatic activity of more than or equal to 500 U/g protein and amyloglucosidase from *Aspergillus niger* with an enzymatic activity of greater than or equal to 300 U/mL were used as catalysts in the enzymatic hydrolysis. Both enzymes were purchased from Sigma-Aldrich (St. Louis, MO, USA), α-amylase acted as the catalyst in liquefaction and amyloglucosidase aided in the saccharification step. Acetic acid (glacial 100%, Merck) and sulfuric acid (Friendemann Schmidt Chemical) were the types of acid used in this experiment.

### Dilute acid pre-treatment

The microalgae were pre-treated with different types and concentrations of acid, where the parameters investigated are types of acid [acetic acid and sulfuric acid] and concentrations of acid [1%, 3%, 5%, 7%, and 9% (v/v)]. A sample without any acid pre-treatment was also prepared and used as the control.

Both of the acids were diluted in conical flasks to concentrations of 1%, 3%, 5%, 7% and 9% (v/v). Distilled water was added first before the acid to prevent the splashing of concentrated acid from the flask due to highly exothermic reaction. Micropipettes and measuring cylinders were used to obtain the accurate volume of concentration. A total of 11 samples with different concentrations of acetic acid and sulfuric acid mixtures were prepared, along with a sample without any acid pre-treatment as the control. All experiments were done in triplicate. Diluted acid with different concentrations were prepared using formula as below:

$$M_{1} V_{1} = \, M_{2} V_{2}$$

where *M*_1_ = concentration in molarity of the concentrated solution, *V*_1_ = volume of the concentrated solution, *M*_2_ = concentration in molarity of the diluted solution, *V*_2_ = volume of the diluted solution.

The microalgae and diluted acid were mixed well in the 200 mL bottle with cap using a scientific multi stirrer, SMHS-3 model for 5 min at 400 rpm to ensure the microalgae powder was completely dissolved. Then, the glass bottles were placed inside the Thermoline drying oven SOV70B model at 120 °C for 30 min. The bottles were cooled down to room temperature before adjusting the pH value of the hydrolysates.

### Enzymatic saccharification

The pH value of the hydrolysates was adjusted to pH 5.5 using NaOH before pre-treatment with the enzymes, α-amylase and amyloglucosidase, for liquefaction and saccharification process, respectively. The liquefaction process was conducted at 90 °C and stroke speed of 120 spm for 120 min. This was followed by the saccharification process with amyloglucosidase at 70 °C for 120 min using a Memmert WNE 45 water bath shaker.

### Yeast cultures media preparation and growth conditions

*Saccharomyces cerevisiae* (*S. cerevisiae*) Type II, which was obtained from Sigma-Aldrich, was selected for the fermentation process in this experiment. The dry yeast was activated by adding 50 mL of distilled water and kept in an incubator at 32 °C for 6 h. Agar solution containing 2 g of yeast extract, 4 g of bacterial peptone, 4 g of glucose and 12 g of yeast peptone dextrose agar in 200 mL distilled water was prepared. The activated yeasts were then grown on petri dish filled with the gelled agar. The yeast cultivation process was conducted in a UV laminar flow chamber to prevent contamination. All samples were prepared under sterilized condition. Then, the cultivated yeasts were kept in an incubator at a temperature of 32 °C for 24 h to oculate it before used for bioethanol production.

### Microalgal fermentation

**Fig. 1:**
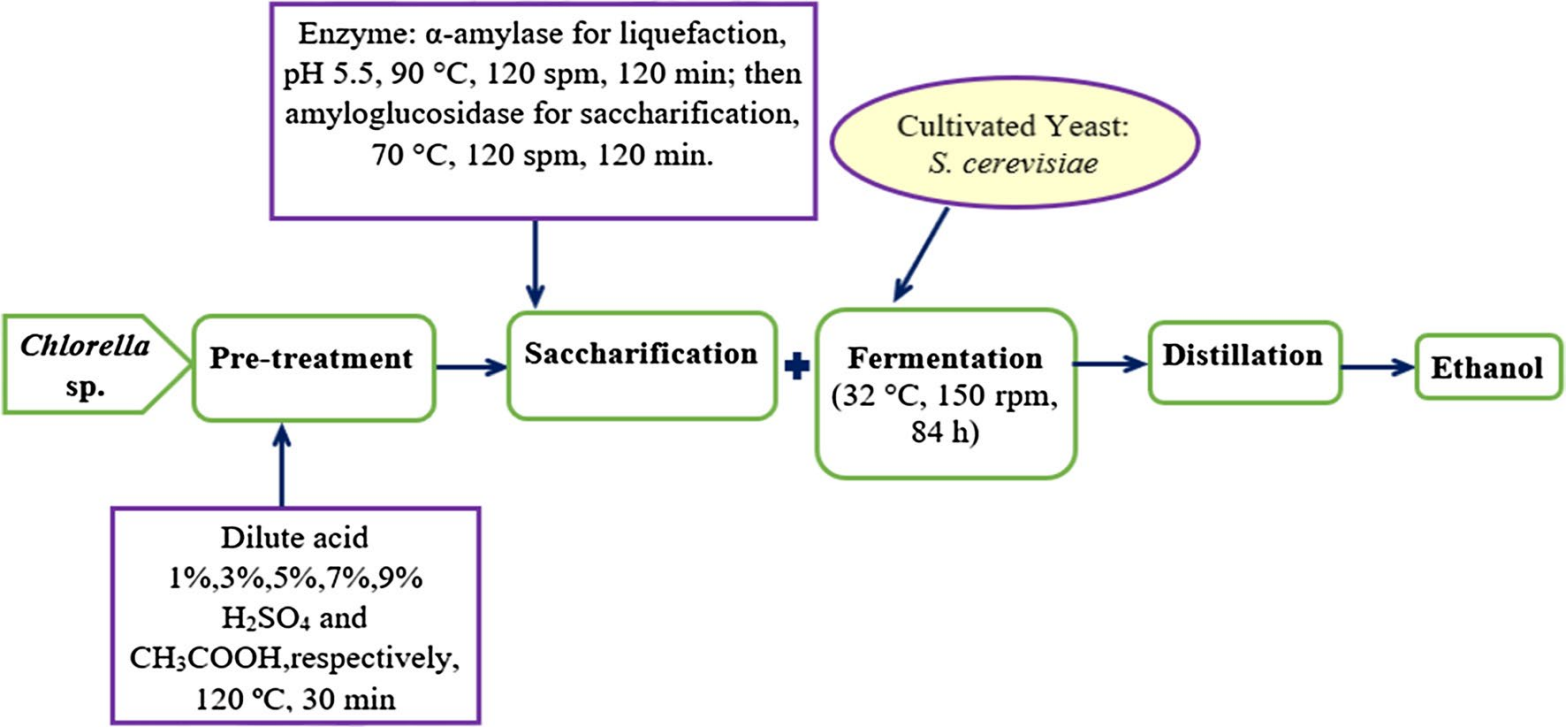
Experimental set up of the fermentation process

Simultaneous saccharification and fermentation (SSF) process was used in this study. 5% (w/v) of cultivated yeast cells, *Saccharomyces cerevisiae* were added in each reagent bottles containing yeast nutrients, 1 g of yeast extract, 0.4 g of potassium dihydrogen phosphate (KH_2_PO_4_) and 0.2 g of ammonium chloride (NH_4_Cl) for every 100 mL of hydrolysate. The fermentation parameters were based on the work by Dahnum et al. with slight modifications [15]. The fermentation process was carried out in a shaking incubator under constant temperature of 32 °C with agitation of 150 rpm for 84 h. Samplings were taken every 12 h to monitor the change in the reducing sugar contents in each samples through DNS method. After 84 h, the ethanol was extracted through distillation process using a rotary evaporator (IKA RV10) under a manual condition of temperature, pressure and rotary speed of 65 °C, 186 bar, and 120 rpm, respectively. All experimental tests were done in triplicates. Flow diagram of the process is shown in Fig. 1.

### Analytical method

#### Reducing sugar concentration

Reducing sugar concentration (g/L) was analysed using 3,5-dinitrosalicylic acid (DNS) method [16]. Samples were taken every 12 h to observe the changes of reducing sugar during the fermentation process. The sample was evaluated by adding 1 ml samples (every 12 h till 84 h) with 100× dilution factor to mixed with 1 mL of DNS reagent. The test tubes with diluted samples and DNS solution were then immersed into water bath shaker for 5 min. The samples were then measured by an UV–Vis spectrometer (SPEKOL^®^ 1500, Analytik Jena, Berlin, Germany) at wavelength at 540 nm by employing a standard curve using glucose to determine the measurement of reducing sugar contents.

#### FTIR spectroscopy

Fourier-transform infrared (FTIR) spectroscopy was used to examine the functional group of alcohols bonds existing in the samples after distillation process. PerkinElmer Spectrum 400 FTIR/FT-FIR Spectrometer with a region of 4000–400 cm^−1^ was used to evaluate the chemical structure of bioethanol from microalgal *Chlorella*.

#### GC-FID

The concentration of the ethanol produced from *Chlorella* was analysed by a gas chromatography equipped with flame ionization detector, GC-FID (Agilent Technologies, 7697A headspace). The analysis was performed using Agilent DB-624 column (30 m × 320 µm × 1.8 µm) with hydrogen as carrier gas at 1 mL/min, and GC conditions of oven temperature: 80 °C, loop temperature: 90 °C and detector temperature: 100 °C. The concentrations of ethanol were quantified with calibration curves modified according to European Standard EN 14110.

#### ANOVA two-way analysis

ANOVA two-way (*p* < 0.05) was performed to determine the relationships of two factors which are concentrations and types of acid to the ethanol contents of the 10 samples from *Chlorella* (*n* = 3 replicates).

#### Determination of ethanol yield

The ethanol yield, *Y*_e_ (g ethanol/g microalgae) was calculated using the equation:

1 $$Y_{e} \, = \,\frac{{V_{\text{D}} C_{\text{EtOH}} \rho_{\text{EtOH}} }}{{m_{B} }}$$

where *V*_D_ is the volume obtained after distillation process (ml), *C*_EtOH_ is the concentration of ethanol determined by GC-FID (%), *ρ*_EtOH_ is the density of ethanol (0.789 g/cm^3^) and *m*_B_ is the weight of the dry biomass (g).

## Results and discussion

### Reducing sugar testing by DNS method

**Fig. 2:**
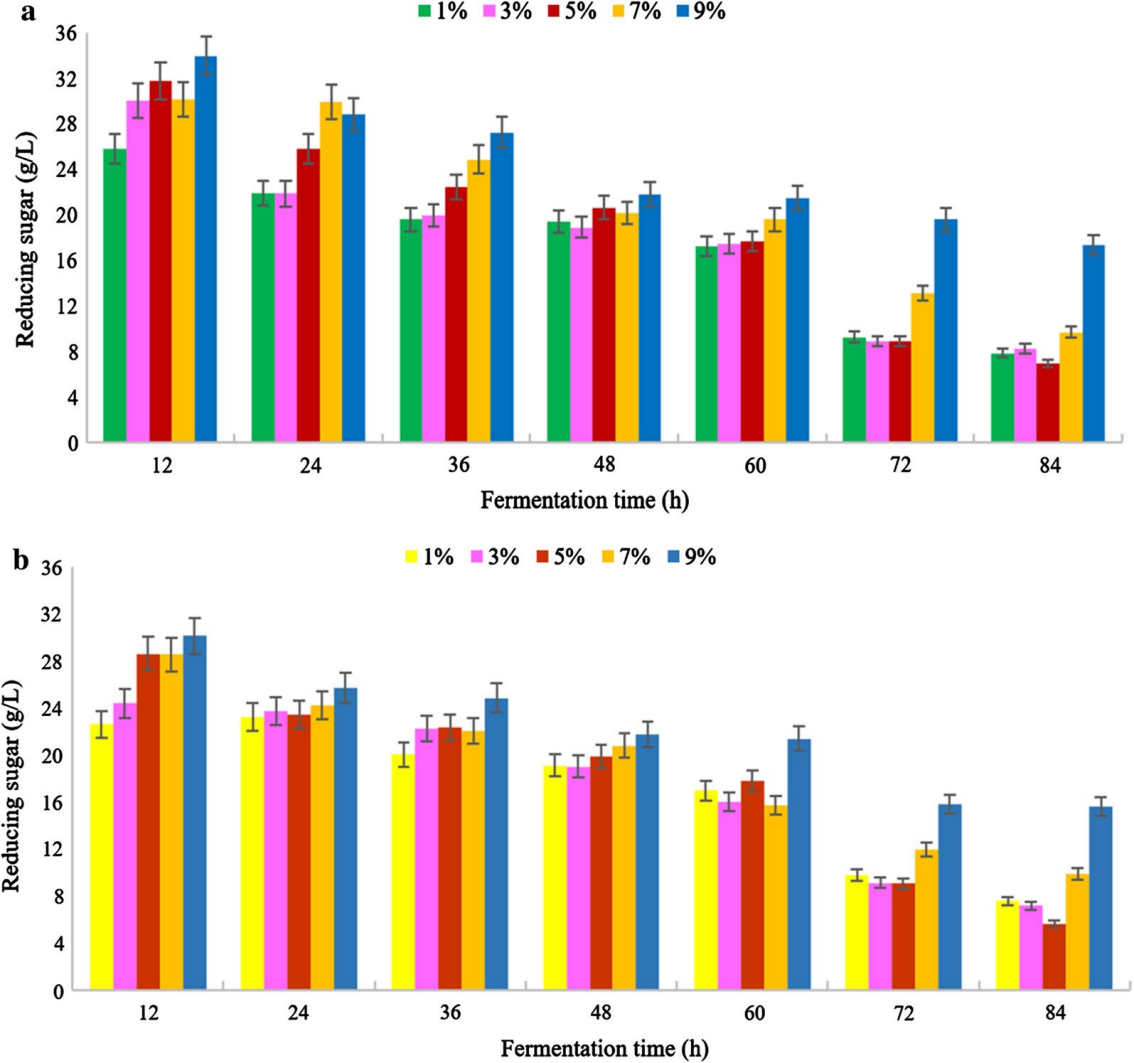
Reducing sugar concentration of *Chlorella* pre-treated by **a** dilute sulfuric acid, H_2_SO_4_ and **b** acetic acid, CH_3_COOH in different concentrations versus fermentation time from 12 to 84 h

**Fig. 3:**
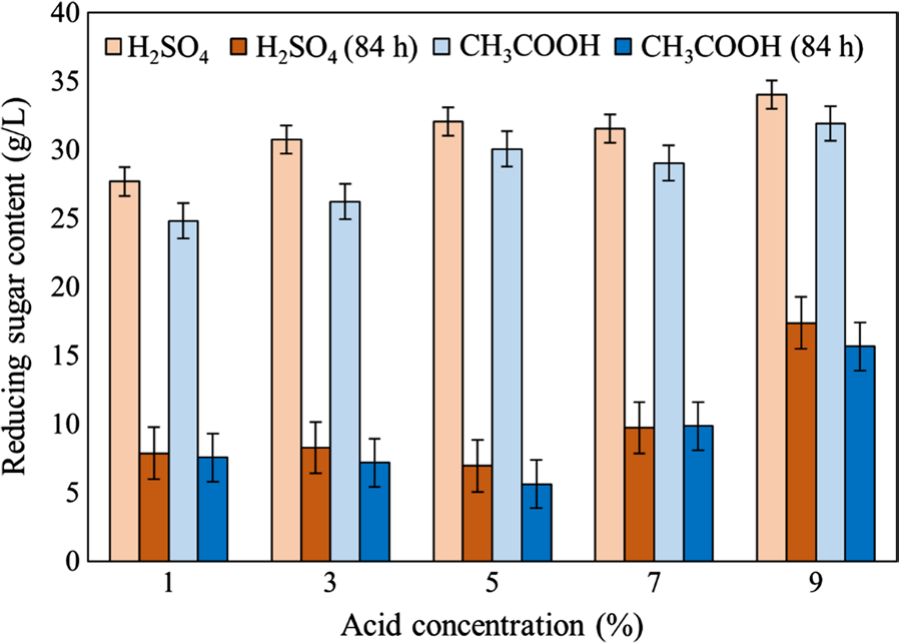
Difference between reducing sugar concentration (g/L) of *Chlorella* after pre-treatment with H_2_SO_4_ and CH_3_COOH and after 84 h fermentation process

Figure 2a, b shows the variation of reducing sugar concentrations in pre-treated microalgal *Chlorella* with sulfuric acid (H_2_SO_4_) and acetic acid (CH_3_COOH) in different time periods during the 84 h fermentation process. In addition, differences of the reducing sugar concentrations were calculated and compared after the fermentation process. As shown in Fig. 3, the reducing sugar content was highly consumed by sample pre-treated with 5% (v/v) H_2_SO_4_ (S5) and 5% (v/v) CH_3_COOH (A5) which was from 32.04 to 6.95 g/L and 30.05 to 5.63 g/L, respectively, after 84 h fermentation process. The control sample showed a reducing sugar concentration of only 20.25–5.63 g/L. Furthermore, the major difference of reducing sugar concentration of S5 and A5 after 84 h fermentation which were 25.09 g/L and 24.42 g/L, respectively. As a result, the pre-treated samples for both sulfuric and acetic acid were comparable. According to Mosier et al. [17], one of the limitations of the dilute mineral acid was high corrosivity compared to organic acid. Hence, acetic acid pre-treatment has more advantages over the sulfuric acid as it achieved better recovery of fermentable sugars and higher recovery process. Even though S9 and A9 both generated the highest reducing sugar concentration after pre-treatment, they obtained the least consumed sugar which was recorded as 16.65 g/L and 16.25 g/L, respectively. This may be due to high concentrations of acid during pre-treatment that converted the monosaccharides to some inhibitors such as furfural, leading to decrease of the fermentable sugars to be consumed [18].

Chng et al. [19] reported that a low concentration of acid with high temperature is desirable than a high concentration acid because it will not cause the degradation of the fermentable sugars to other unfavorable compounds which affects the hydrolysis yield. Likewise, a low concentration of acid pre-treatment is more considerable than a high concentration acid due to lesser amounts of neutralizing agent being needed in the following steps and there will be less tendency to corrode the experimental equipment. Dilute acid hydrolysis is more cost effective and has been known as a feasible method to produce bioethanol from carbohydrate-rich microalgae biomass.

### FTIR characterization

**Fig. 4:**
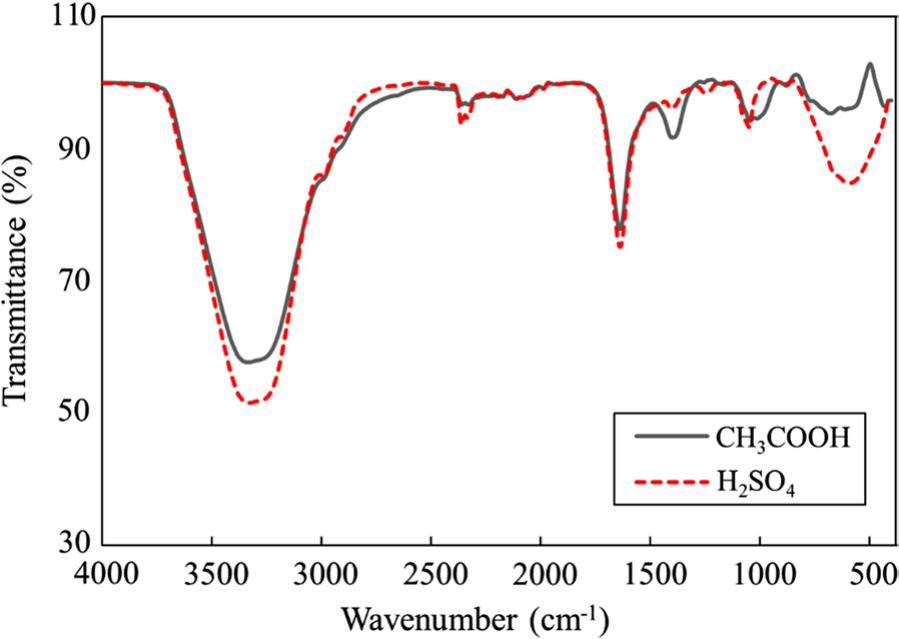
FTIR spectroscopy analysis illustrates the comparison of bioethanol produced from *Chlorella* with pre-treatment by H_2_SO_4_ and CH_3_COOH

FTIR results obtained in this study showed some common peaks in both bioethanol from microalgal *Chlorella* pre-treated with acetic and sulfuric acid [20, 21]. The absorption spectrum in Fig. 4 shows peaks between the ranges of 3400–3200 cm^−1^ (hydroxyl group), 2356–2322 cm^−1^, 1658–1638 cm^−1^ (alkene group), 1384–1377 cm^−1^, and 1060–1001 cm^−1^ (ethanol and glucose). By comparing the FTIR results obtained in this research with other studies, Kassim and Bhattacharya [20] reported that the peak between 3400 and 3200 cm^−1^ represents the hydroxyl (OH) group in the samples. Moreover, the presence of absorption wave between 1658 and 1638 cm^−1^ represents an existence of alkene group with variable C=C bonds between atoms with medium intensity [22]. Veale et al. [23] reported that peak between 1060 and 1001 cm^−1^ was a reflection of ethanol and glucose found in the wave regions between 1200 and 800 cm^−1^ due to the absorptions bands of C–O and C–C stretch vibration. Furthermore, the peak at 1100–900 cm^−1^ indicated the presence of the carbohydrates [24]. Therefore, FTIR analysis was able to quantify the functional group existing in the bioethanol produced from microalgae.

### GC-FID ethanol concentration analysis

**Fig. 5:**
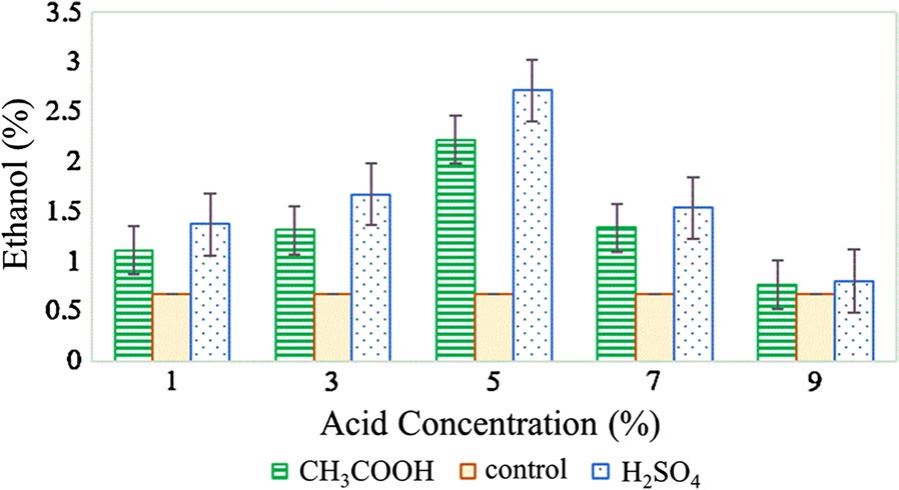
Concentration of ethanol detected by GC-FID

Figure 5 shows the concentration of ethanol that was identified by GC-FID. The highest concentration of ethanol was found in the sample S5 (2.71%) followed by A5 (2.22%). To compare the effect of pre-treatment bioconversion, an untreated control sample was subjected to the fermentation process. An ethanol concentration of 0.68% was found in the untreated control sample. This result was much lower than those pre-treated samples with acid hydrolysis. Hence, the use of pre-treatment is critical before fermentation process to increase the ethanol productivity [25]. Moreover, the lowest ethanol concentration in sulfuric acid was 0.77% (S9) while in acetic acid it was 0.80% (A9), both having quite comparable values and are at the highest acid concentration used. Whereas, A1 and S1 both obtained an ethanol concentration of 1.11% and 1.37%, respectively, despite having the lowest acid concentration. This could be due to the lower acid concentration pre-treatment which helps to enhance the downstream fermentation by releasing essential nutrients [26].

### ANOVA two-way analysis of ethanol concentration

The results in Table 1 indicates a statistically significant (*p* < 0.05) difference between effect of the two factors which are concentration (*p* = 0.025) and type of acid (*p* = 0.001) to ethanol contents of the 10 bioethanol samples produced from microalgal *Chlorella*. Miranda et al. [27] reported that a higher concentration of the acid pre-treatment led to the degradation of the fermentable sugars and conversion to other unwanted products. This means that the concentration and the type of acid play a significant role in pre-treatment that will subsequently affect the ethanol productivity.

**Table 1:** Two-way ANOVA analysis of two main factors (concentration of acids and types of acids) related to the ethanol concentration tested via GC-FID

Source	Dependent variable	SS	*Df*	MS	*F*-ratio	*P*-value
Concentration of acid	Ethanol	0.182	1	0.182	7.709	0.025*
Type of acids	Ethanol	3.033	4	0.758	6.388	0.001*

* Significant value *p* < 0.05

### Determination of ethanol yield

Table 2 shows the calculation of ethanol yield (g ethanol/g dry microalgae). Among the samples, the highest ethanol yield 0.28 g/g was found at the S5 followed by 0.23 g/g obtained by A5. The ethanol yield of the untreated control sample was only 0.07 g/g. In fact, Babujanarthanam and Kavitha [25] reported the corresponding highest ethanol yield produced from red algal *Gelidiella acerosa* after using diluted acid and enzymatic pre-treatments was 0.21 g ethanol/g red algae while the untreated red algae was obtained 0.05 g/g of ethanol yield. The result of ethanol yield for the pre-treated and untreated microalgae in this experiment showed slightly higher yield compared to the ethanol produced by *Gelidiella acerosa*. Likewise, there are some typical bioethanol production yields through fermentation from microalgal feedstock using *S. cerevisiae* which have been reported that do not exceed 0.30 g ethanol/g dry algae [12, 28, 29].

**Table 2:** Calculation of ethanol yield (g/g) of acetic and sulfuric acid in various concentrations after distillation

Acid concentration (%, v/v)	Ethanol yield (g/g)		
	CH_3_COOH	H_2_SO_4_	Control
0^a^	N/A	N/A	0.068^a^
1	0.110	0.137	N/A
3	0.133	0.169	
5	0.230	0.281	
7	0.134	0.154	
9	0.080	0.084	

^a^Control without any pre-treatment was used to monitor and compare the pre-treated samples

The possibility for lower ethanol yield in acetic acid with 9% (v/v) concentration (0.08 g ethanol/g biomass) and sulfuric acid with 9% (v/v) concentration (0.08 g ethanol/g biomass) is most likely due to the existence of high concentration of fermentation inhibitors such as formic acid, levulinic acid, and 5-hydroxymethylfurfural (HMF). The formation of these inhibitors during hydrolysis pre-treatment led to a negative impact on the growth of fermentative organisms and indirectly affected the ethanol productivity.

## Conclusion

The study indicated that the types and concentrations of acid used during pre-treatment of microalgae can significantly affect the release of fermentable sugars and the resulting ethanol productivity. This work provides essential information for the potential to replace a strong acid with a weaker acetic acid for chemical hydrolysis due to its effectiveness in releasing fermentable sugars for bioethanol production. The advantages in using acetic acid in pre-treatment are the generation of less chemical discharge and less corrosion towards equipment. Hence, acetic acid pre-treatment can contribute to a more cost-effective and energy-efficient bioethanol production from microalgae.

## Data Availability

Not applicable.
